# Respiratory Syncytial Virus Activity — United States, July 2011–January 2013

**Published:** 2013-03-01

**Authors:** National Respiratory and Enteric Virus Surveillance System laboratories, Amber K. Haynes, Mila M. Prill, Marika K. Iwane

**Affiliations:** Div of Viral Diseases, National Center for Immunization and Respiratory Diseases, CDC

Respiratory syncytial virus (RSV) causes lower respiratory infections among infants and young children worldwide (1). During 1997–2006, an estimated 132,000–172,000 children aged <5 years were hospitalized for RSV infection annually in the United States (2). In temperate climate zones, RSV generally circulates during the fall, winter, and spring (3), but the exact timing and duration of RSV seasons vary by region and year. To determine seasonal trends in the circulation of RSV at national and regional levels, data collected by the National Respiratory and Enteric Virus Surveillance System (NREVSS) were analyzed. For 2011–12, the RSV season onset ranged from late October to mid-January and season offset ranged from early March to early May in all 10 U.S. Department of Health and Human Services (HHS) regions, excluding Florida. Florida is reported separately because it has an earlier season onset and longer duration than the rest of the country. For data reported as of January 7, 2013, RSV onset for the 2012–13 season occurred in all but one of the HHS regions by December 15, 2012. Seasonal patterns remained consistent with previous years and demonstrated the usual differences in RSV circulation among HHS regions. Health-care providers and public health officials can use information on RSV circulation to guide diagnostic testing and timing of RSV immunoprophylaxis for children at high risk for severe respiratory infection.

NREVSS records U.S. laboratory-based specimen data on RSV and other viral pathogens. Each week, participating laboratories voluntarily report weekly aggregated results of RSV tests. For consistency, only results of antigen detection methods are included in the analysis. Antigen detection was used by 94.1% of participating laboratories during 2011–12. Season onset, offset, duration, and peak* are reported for each HHS region,† the state of Florida, and nationally, with and without Florida. This allows geographic variation in RSV activity to be described and accommodates the unusually early and sometimes long RSV season observed in Florida (3).

During July 2011–June 2012, a total of 522 laboratories reported at least 1 week of RSV testing by any detection method to NREVSS. CDC limited this analysis to 174 (33.3%) laboratories in 42 states that met the following criteria: 1) reported RSV antigen testing results for ≥30 weeks during the NREVSS season and 2) averaged ≥10 tests per week during the NREVSS season. Qualifying laboratories reported a total of 270,441 tests, of which 41,299 (15.3%) were positive. Nationally, RSV onset occurred the week ending November 19, 2011, and lasted 21 weeks, until the week ending April 7, 2012 (Table). The proportion of specimens positive for RSV by antigen detection reached a season high of 26.2% during the week ending January 28, 2012. With Florida excluded, the national onset occurred 1 week later (November 27, 2011), and the season duration decreased by 1 week. Onset for the 10 HHS regions (excluding Florida) ranged from late October to mid-January, and season offset ranged from early March to early May. The season peak ranged from mid-January to mid-March, and the duration ranged from 14–23 weeks, with a median of 19 weeks. Region 7 had the shortest season and Region 3 had the longest. The season onset for Florida occurred the week ending August 13, 2011, and the season continued through the week ending March 3, 2012.

What is already known on this topic?

In the United States, respiratory syncytial virus (RSV) begins circulating in the fall, peaks in the winter, and ends during spring. A network of U.S. laboratories reports results of specimens tested for RSV to the National Respiratory and Enteric Virus Surveillance System, which summarizes national, regional, and state-level RSV activity.

What is added by this report?

For the 2011–12 season, RSV circulation began nationally in mid-November and ended in early April. Circulation peaked at 26% of tests positive in late January. During the 2012–13 RSV season, onset occurred in all but one of the 10 U.S. Department of Health and Human Services regions by December 15, 2012. These patterns in national RSV circulation were similar to those observed previously. Onset, offset, and duration varied among the regions and Florida.

What are the implications for public health practice?

RSV surveillance alerts public health officials and clinicians to times when respiratory infections might be attributed to RSV and when patients at high risk for severe complications of infection might need RSV immunoprophylaxis.

The 2012–13 RSV onset analysis is limited to laboratories that reported results for at least 1 week of the NREVSS season and at least one antigen test on average per week during the NREVSS season. Preliminary analysis of these data included a total of 135,849 RSV antigen tests and 19,903 (14.7%) positive results reported by 462 eligible laboratories from the 50 states and the District of Columbia. The season onset occurred in nine of the 10 HHS regions by December 15, 2012. As of January 7, 2013, onset had not occurred in Region 8, but additional cases were being reported in other regions.

Nationally, RSV onset occurred the week ending October 27, 2012; however, when Florida is excluded from analysis, the national onset occurred 1 week later (week ending November 10, 2012) (Table). Weekly updates of RSV national, regional, and state RSV trends are available from NREVSS at http://www.cdc.gov/surveillance/nrevss. Additional information regarding Florida RSV trends is available from the Florida Department of Health at http://www.doh.state.fl.us/disease_ctrl/epi/rsv/rsv.htm.

## Editorial Note

During July 2011–June 2012, national and regional RSV trends were similar to patterns previously reported for 2010–11. Florida’s season onset occurred 5 weeks earlier than the previous season, and each HHS region differed in onset, offset, and duration. Florida’s earlier onset has been well documented, as have differences in activity from year-to-year in the same geographic location (3). Social and demographic factors, population density, pollution, and climate each might influence RSV activity (3–6).

NREVSS surveillance data can be used to identify RSV activity and coordinate timing of RSV immunoprophylaxis with palivizumab. Palivizumab is a monoclonal antibody against RSV recommended by the American Academy of Pediatrics (AAP) to be administered to children at high risk for severe RSV disease (7). AAP also provides guidelines for identifying infants and young children likely to benefit from immunoprophylaxis (e.g., certain infants with congenital heart disease or chronic lung disease, and those born prematurely) and for timing of RSV immunoprophylaxis by region (7). NREVSS provides timely data on RSV activity at the national, regional, and state levels, which have been correlated with numbers of RSV-associated hospitalizations in select regions (8). Consequently, health-care providers and public health officials use NREVSS data to guide diagnostic testing and to assess possible causes of regional respiratory infection outbreaks.

The findings in this report are subject to at least four limitations. First, reporting to NREVSS is voluntary and might be biased to more active reporters. Second, the percent positive detections reflect not only disease burden (i.e., number of cases per capita or severity of seasonal outbreaks) but also the volume of tests ordered. Third, although NREVSS data can be used to approximate regional RSV seasonal characteristics, they cannot be used to estimate RSV activity in every state or county because participation varies from year-to-year and between states. Finally, periods of low RSV activity might not be captured by the NREVSS onset and offset definitions. Despite these limitations, NREVSS provides useful guidance to physicians ordering diagnostic tests and planning to initiate immunoprophylaxis.

## Figures and Tables

**TABLE:** Summary of 2011–12 respiratory syncytial virus season and 2012–13 season onset, by U.S. Department of Health and Human Services (HHS) Region* and Florida† — National Respiratory and Enteric Virus Surveillance System, June 2011–January 2013

	2011–12 season					2012–13 season	
		
HHS region or state	No. of laboratories reporting	Onset week ending	Peak week ending	Offset week ending	Season duration (wks)	No. of laboratories reporting	Onset week ending
**National**	**174**	**11/19**	**1/28**	**4/7**	**21**	**462**	**10/27**
National without Florida	156	11/26	1/28	4/7	20	432	11/10
Florida	18	8/13	12/3	3/3	30	30	7/21
Region 3	17	10/22	1/7	3/24	23	52	10/27
Region 2	17	11/12	12/17	3/17	19	28	11/3
Region 6	30	11/19	1/28	3/31	20	58	10/27
Region 1	6	12/3	1/7	3/10	15	27	11/24
Region 4§	20	12/3	12/31	3/31	18	66	11/10
Region 5	22	12/10	3/17	4/28	21	72	11/24
Region 9	19	12/17	2/25	5/5	21	46	11/3
Region 10	8	12/24	3/3	4/21	18	25	12/15
Region 8	7	1/7	2/18	5/12	19	28	—¶
Region 7	10	1/14	3/17	4/14	14	30	11/24

* Ranked by 2011–12 onset week ending date. Listed with headquarters city for each region; territories not included. *Region 1* (Boston): Connecticut, Maine, Massachusetts, New Hampshire, Rhode Island, and Vermont; *Region 2* (New York): New Jersey and New York; *Region 3* (Philadelphia): District of Columbia, Delaware, Maryland, Pennsylvania, Virginia, and West Virginia; *Region 4* (Atlanta): Alabama, Florida, Georgia, Kentucky, Mississippi, North Carolina, South Carolina, and Tennessee; *Region 5* (Chicago): Illinois, Indiana, Michigan, Minnesota, Ohio, and Wisconsin; *Region 6* (Dallas): Arkansas, Louisiana, New Mexico, Oklahoma, and Texas; *Region 7* (Kansas City): Iowa, Kansas, Missouri and Nebraska; *Region 8* (Denver): Colorado, Montana, North Dakota, South Dakota, Utah, and Wyoming; *Region 9* (San Francisco): Arizona, California, Hawaii and Nevada; and *Region 10* (Seattle): Alaska, Idaho, Oregon, and Washington. Maine, Rhode Island, Vermont, New Mexico, Nebraska, Utah, Wyoming, and Idaho did not have any participating laboratories in the 2011–12 season analysis.

† Florida is reported separately because it has an earlier onset and longer duration than other states.

§ Excludes data from Florida.

¶ As of January 7, 2013, the 2012–13 season onset had not occurred.

## Footnotes

* In NREVSS, the onset week in an area (national, regional, or state) is defined as the first of 2 consecutive weeks when the weekly mean of the percentages of specimens testing positive for RSV antigen in all reporting laboratories in the area is ≥10%. The offset is the last of 2 consecutive weeks when the mean percent positive drops below this threshold. The season duration is the onset week, the weeks between onset and offset, and the offset week. The peak is the week when the mean percentage of positive RSV antigen tests is the highest.

† Listed with headquarters city for each region; territories not included. *Region 1* (Boston): Connecticut, Maine, Maryland, New Hampshire, Rhode Island, and Vermont; *Region 2* (New York): New Jersey and New York; *Region 3* (Philadelphia): Delaware, District of Columbia, Maryland, Pennsylvania, Virginia, and West Virginia; *Region 4* (Atlanta): Alabama, Georgia, Kentucky, Mississippi, North Carolina, South Carolina, and Tennessee; *Region 5* (Chicago): Illinois, Indiana, Michigan, Minnesota, Ohio, and Wisconsin; *Region 6* (Dallas): Arkansas, Louisiana, New Mexico, Oklahoma, and Texas; *Region 7* (Kansas City): Iowa, Kansas, Missouri, and Nebraska; *Region 8* (Denver): Colorado, Montana, North Dakota, South Dakota, Utah, and Wyoming; *Region 9* (San Francisco): Arizona, California, Hawaii and Nevada; *Region 10* (Seattle): Alaska, Idaho, Oregon, and Washington. Maine, Rhode Island, Vermont, New Mexico, Nebraska, Utah, Wyoming, and Idaho did not have any laboratories that met the inclusion criteria for the 2011–12 season analysis.
